# Systematic review and meta‐analysis of the association between childhood physical activity and age at menarche

**DOI:** 10.1111/apa.14711

**Published:** 2019-01-15

**Authors:** Lucia Calthorpe, Soren Brage, Ken K. Ong

**Affiliations:** ^1^ Department of Public Health and Primary Care University of Cambridge Cambridge UK; ^2^ School of Medicine University of California at San Francisco San Francisco CA USA; ^3^ MRC Epidemiology Unit Institute of Metabolic Science University of Cambridge Cambridge UK; ^4^ Department of Paediatrics University of Cambridge Cambridge UK

**Keywords:** Age at menarche, Athlete, Physical activity, Puberty timing

## Abstract

**Aim:**

To systematically appraise and summarise published evidence on the association between childhood physical activity (PA) and subsequent age at menarche (AAM).

**Methods:**

We searched PubMed (1990–2018) for studies that reported the relationship between childhood PA and AAM. We performed tabular synthesis of population‐based studies and a random‐effects meta‐analysis of results of athlete/nonathlete studies.

**Results:**

One randomised controlled trial was identified, in which an intervention to prevent obesity reduced the likelihood of menarche during the two‐year study period (relative risk: 0.75, 95% CI: 0.66–0.87; n = 422 girls). One of five prospective cohort studies (total n = 4492) reported a significant association between self‐reported PA duration and subsequent menarche timing. Four of five historical cohort studies (total n = 89 470) reported significant associations between recalled premenarcheal PA and later AAM. Meta‐analysis across 12 athlete/nonathlete studies showed that menarche occurred 1.13 years later (95% CI: 0.80–1.47) in athletes compared to nonathletes.

**Conclusion:**

These findings suggest that AAM is a behaviourally modifiable trait. However, the quality of reported population‐based study evidence is low and estimation of the true relationship between childhood PA and AAM is likely confounded by concomitant changes in diet and lifestyle behaviours.

**Abbreviations**
AAM: Age at menarche
BMI: Body mass index
HCS: Historical cohort study
PA: Physical activity
PCS: Prospective cohort study
RCT: Randomised control trial

**Key Notes**

- This systematic literature review found that the only randomised controlled trial and five of ten cohort studies reported a significant association between premenarcheal physical activity and later age at menarche (AAM), a key measure of puberty timing in girls.
- Meta‐analysis across 12 athlete/nonathlete studies showed that menarche occurred 1.13 years later in athletes compared to nonathletes.
- These findings suggest that AAM may be a behaviourally modifiable trait.

## Introduction

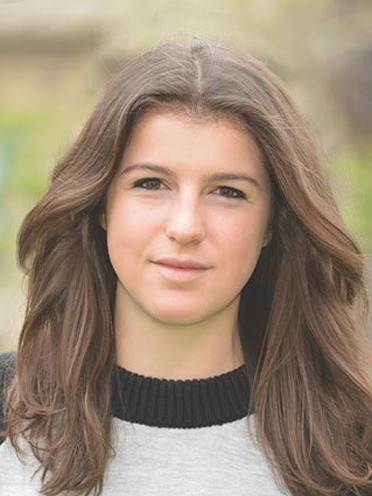

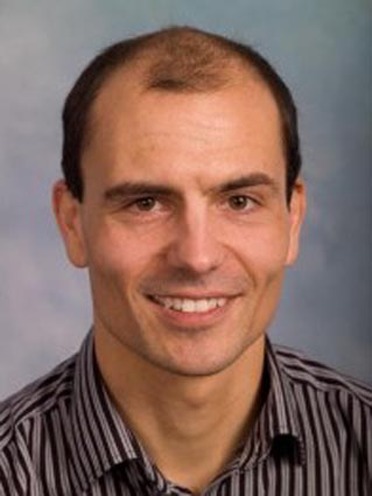

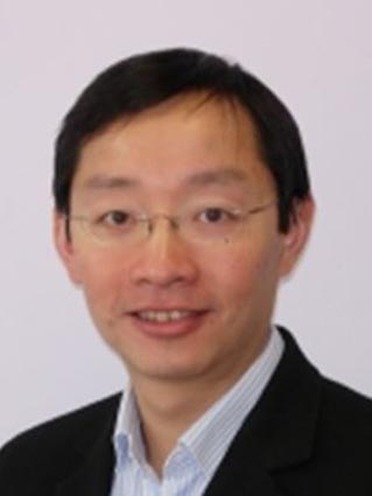

Puberty, the process of rapid growth and sexual maturation that separates childhood from adulthood, represents a critical period in normal development. The timing of puberty has been associated with a wide range of later health outcomes. In general, earlier puberty timing is associated with higher risks of adverse health and behavioural outcomes. Short‐term outcomes include depression, substance abuse, earlier age at first sexual intercourse, suicide and eating disorders 1, 2, 3. Long‐term outcomes include cardiovascular disease, Type 2 diabetes, breast cancer and all‐cause mortality 4, 5, 6, 7.

Age at menarche (AAM), a young woman's first menstrual bleed, is a widely used measure of puberty timing because it is a distinct and notable event that is usually well‐recalled 8, 9, 10. Widely available data on AAM have been used to demonstrate population‐level secular trends towards younger AAM that appear to parallel improvements in socio‐economic conditions. In the UK, such secular trends were observed over most of the 20th century and are still detectable in recent birth cohorts 11.

Puberty timing is a multifactorial trait, and a wide range of genetic and environmental determinants have been proposed. Large‐scale genetic studies have identified nearly 400 independent genetic variants that are robustly associated with AAM, a trait that clusters within families and has an estimated heritability of 50–70% 12. AAM has also been associated with several potentially modifiable factors, including prenatal exposures, birthweight, childhood nutrition and body mass index (BMI), socio‐economic circumstances and stress and physical activity (PA) 13, 14. While delayed puberty in elite athletes has been documented, less attention has been paid to the potential role of moderate PA levels. For example, Yermachenko and Dvornyk's systematic review of nongenetic determinants of AAM identified five papers which demonstrated a potential relationship between high‐intensity exercise, largely in combination with nutritional deprivation, and delayed AAM. However, these authors did not explicitly include ‘PA’ or a similar term in their search 14. Here, we aimed to systematically review the association between PA and AAM, by including findings from both population‐based cohorts and athlete versus nonathlete studies.

## Methods

### Literature search

To identify papers reporting on the relationship between childhood (specifically premenarcheal) PA and subsequent AAM, we conducted a literature search in Medline (Ovid) up to January 25, 2017. The search terms were chosen to be inclusive:

[female*.mp OR girl*.mp] AND

[exp Exercise/OR exercis*.mp. OR physical activit*.mp. OR physical activity.mp] AND

[exp Puberty, Precocious/OR exp Menarche/OR menarch*.mp. OR exp Puberty/OR exp Sexual Maturation/or sexual maturation*.mp. OR sexual maturity*.mp. OR pubertal*.mp]

**Figure 1:**
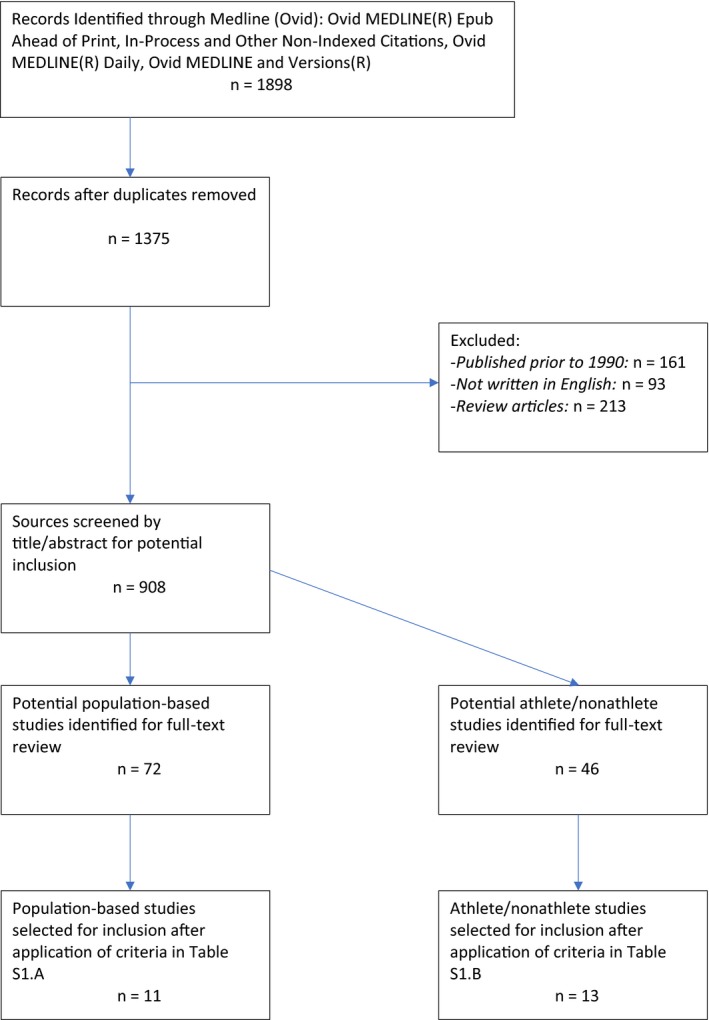
Search strategy flow chart.

After exclusion of duplicates, papers published prior to 1990 (in the light of the secular changes in puberty timing), non‐English language papers and reviews without primary data, we screened the title and abstract of 908 papers for relevance (Fig. 1). We identified 72 papers describing population‐based cohorts and 46 papers on athlete/nonathlete studies to be of potential relevance, and these were reviewed in full text against the exclusion criteria (Tables S1 and S2). Given that early menarche has been shown to lead to subsequent decline in PA 15, 16, 17, we excluded cohort studies that did not assess PA *prior* to menarche. Among athlete/nonathlete studies, we included studies of dancers as a subtype of athlete because of comparable duration and intensity of dance PA. The search was updated on September 7, 2018, but no further relevant studies were identified.

Study quality was assessed using the NIH Quality Assessment Tool for Observational Cohort and Cross‐Sectional Studies.

### Meta‐analysis

From athlete/nonathlete studies, we extracted data on the between‐group difference in AAM. For those studies that did not report this difference, group means and standard deviations for AAM were converted to mean difference (μ_1_‐μ_2_) and standard error for the difference. Inverse‐variance‐weighted random‐effects meta‐analysis was conducted using the *metan* command in Stata v14.2 (StataCorp LP, College Station, TX, USA).

## Results

### Population‐based studies

Population‐based studies were further classified by study design: (i) randomised control trials (RCT; n = 1); (ii) prospective cohort studies (PCS; n = 5), which assessed PA in premenarcheal girls who were then followed to menarche, or (iii) historical cohort studies (HCS; n = 5), in which PA prior to menarche was recalled in postmenarcheal girls (Tables S2 and S3). Study quality was rated as higher among PCS (range: 7–12) than among HCS (range: 5–7) (Tables S4 and S5). All studies assessed PA using self‐reported measures.

The one identified RCT reported that a school‐based intervention (‘Planet Health’), designed to prevent obesity in premenarcheal girls (age range 10–13 years old; n = 422), reduced the likelihood of menarche occurring during the two‐year intervention period (RR = 0.75, 95% CI: 0.66–0.87) 18. PA was estimated to account for roughly 1/3 of the intervention effect (RR adjusted for PA = 0.84, 95% CI: 0.74, 0.96). The effect of the intervention on menarche was further attenuated when changes in screen time, BMI and triceps skinfold thickness were controlled for 18.

The five PCS covered diverse geographical areas, ranged in size from n = 167 to n = 2487 girls and used a variety of measures of PA (Table 1; Table S2). Weight‐adjusted energy expenditure was associated with lower risk of menarche (i.e. later menarche) in one study (Merzenich et al.), but two other studies found no association (table 6 19, 20, 21, 22). A fourth study (Koo et al.) reported a higher risk of menarche associated with energy expenditure (i.e. earlier menarche) but did not adjust for body weight 21. PA duration was associated with lower risk of menarche in one study (Merzenich et al.), but one other study found no association (Moisan et al.) 19, 22. One study (Tehrani et al.) examined a dichotomised measure of PA and found similar mean AAM between active and nonactive girls 23.

**Table 1:** Summary of associations between physical activity and menarche timing: prospective cohort studies

First author (citation)	Country, Year published	Sample size	Dichotomised PA groups	PA duration	Energy expenditure
Moisan 19	Canada, 1991	n = 2487		Incidence Ratio (95% CI): Menarche, 2 years FU. Ref = Quartile 1 Quartile 2: 0.9 (0.8–1.1) Quartile 3: 1.0 (0.8–1.2) Quartile 4: 1.0 (0.8–1.1) p‐trend = 0.8, adjusted for age, mother's AAM	Incidence Ratio (95% CI): Menarche, 2 years FU. Ref = Quartile 1 Quartile 2: 1.1 (0.9–1.3) Quartile 3: 1.1 (0.9–1.3) Quartile 4: 1.0 (0.8–1.2) p‐trend = 0.8, adjusted for age, mother's AAM, weight
Merzenich 22	Germany, 1993	n = 167		Risk Ratio (95% CI): Menarche, 2 years FU. Ref = Quartile 1 Quartile 2: 0.7 (0.42–1.22) Quartile 3: 0.4 (0.21–0.69) Quartile 4: 0.3 (0.12–0.51) p‐trend <0.0001, adjusted for age, fat intake, per cent body fat	Risk Ratio (95% CI): Menarche, 2 years FU. Ref = Quartile 1 Quartile 2: 0.8 (.47–1.30) Quartile 3: 0.7 (0.40–1.09) Quartile 4: 0.6 (.34–0.98) p‐trend = 0.03, adjusted for age, weight (p = 0.8 with 7‐day diary data)
Chie 20	Taiwan, 1997	n = 799			Odds Ratio (95% CI): Menarche, 1 years FU. Ref = <2000 kcal/wk 2000–3999 kcal/wk: 1.0 (0.5–2.0) >4000 kcal/wk: 0.4 (0.2–1.0) p‐trend = 0.098, adjusted for age, weight
Koo 21	Canada, 2002	n = 637			Hazard Ratio (95% CI): Menarche, 3 years FU, Ref = Quartile 1 Quartile 2: 1.10 (0.69–1.74) Quartile 3: 1.45 (0.93–2.27) Quartile 4: 1.46 (0.95–2.24) p‐trend = 0.039, adjusted for age only
Tehrani 23	Iran, 2014	n = 402	AAM mean (SD), year Active: 13.08 (1.29) Passive: 12.99 (1.12) p‐value not stated		

PA = Physical activity, AAM = Age at menarche, FU = Follow‐up, Ref = Reference group.

The five HCS were mostly European, and sample sizes ranged from 750 to 81 438 girls (Tables 2 and S3). PA duration was associated with later AAM in all three studies that reported this measure, although effect estimates differed widely 24, 25, 26. Two studies tested dichotomised PA exposures: one study (Vandeloo et al.) reported that more active girls had significantly later AAM, but the other study found no association (Papadimitriou et al.) 27, 28.

**Table 2:** Summary of associations between physical activity and menarche timing: historical cohort studies

First author (citation)	Country, Year published	Sample size	Dichotomised PA groups	PA duration
Veronesi 26	Italy, 1994	n = 2930		Mean Difference (SE): AAM (years) Ref: Moderate PA (2 h/wk) Regular: 0.35 (0.144) Intense: 0.66 (0.206) p‐ANOVA <0.01
Chavarro 25	Colombia, 2004	n = 3206		Mean difference (95% CI): AAM (years) Ref: Not physically active: <1 h/d: 0.05 (‐0.04, 0.15) 1–2 h/d: 0.07 (‐0.08, 0.2) >2 h/d: 0.28 (0.06, 0.50) p‐trend = 0.02, adjusted for Place of birth, Maternal education, Socio‐economic status, Family size, Year of birth, Year of interview
Vandeloo 27	Belgium, 2007	n = 1146	No sport vs. Some sport (Ref) Hazard Ratio (95% CI): Time to menarche (year) 1.28 (1.054, 1.573) p = 0.013	
Papadimitriou 28	Greece, 2008	n = 750	Active vs. Passive Mean difference (SE), AAM (year) 0.09 (0.09) *T*‐test: p = 0.3	
Morris 24	UK, 2010	n = 81 438		Linear regression coefficient (95% CI): AAM (years) 0.0983 (0.065, 0.133) per 10 h/wk PA p < 0.01, adjusted for Ethnicity, Weight, Height, Number of siblings, Birth order, Maternal age, Twinning, Exposure to preeclampsia, Birthweight

PA = Physical activity, AAM = Age at menarche, Ref = Reference group.

### Athlete versus nonathlete studies

Thirteen studies identified athletes (defined by their participation prior to menarche) and compared these to nonathletes with regard to AAM, which was assessed prospectively in two studies and retrospectively in 11 studies (Table S6). Between‐group differences in AAM were meta‐analysed across studies using random‐effects models, excluding one study (Schevchenko et al.), which did not report standard errors or standard deviations for AAM 29.

**Figure 2:**
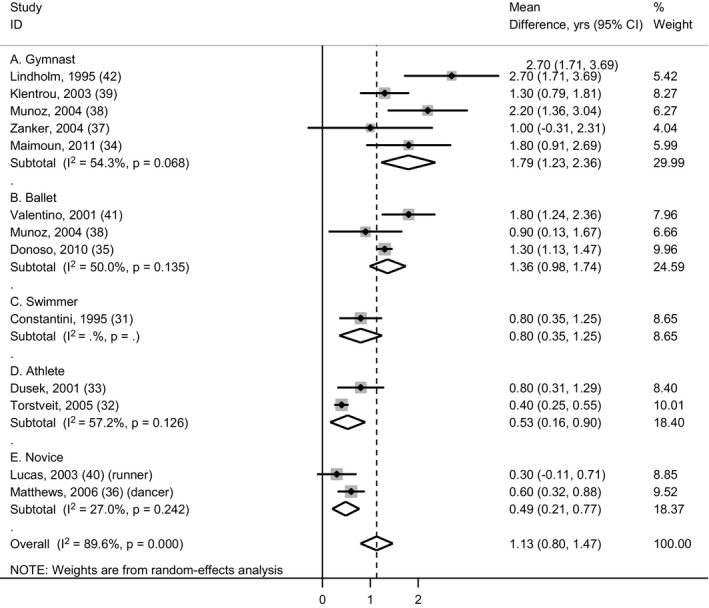
Forest plot showing mean differences in age at menarche (years) in athletes compared to nonathletes, categorised by type of athlete. Data are from inverse‐variance‐weighted random‐effects meta‐analysis.

Overall, menarche occurred 1.13 years (95% CI: 0.80–1.47) later in athletes compared to nonathletes (Fig. 2). Mean differences ranged from 1.79 years (1.23–2.36; five studies) later in gymnasts compared to nongymnasts to 0.49 years (0.21–0.77; two studies) later in novice (nonelite) athletes compared to nonathletes. High heterogeneity was observed in effect estimates between studies (I‐squared: 89.6%), but this was less within subgroups of studies of the same athlete type (I‐squared: 50.0–57.2%).

## Discussion

We identified a large number of studies (n = 24) reporting on the possible relationship between PA and AAM, however, most used study designs that are considered to provide relatively low levels of evidence. All 13 athlete/nonathlete studies reported a difference in AAM, 4/5 HCS reported a significant association between recalled premenarcheal PA and later AAM, but only 1/5 PCS reported a significant association between premenarcheal PA and later timing of menarche. The one RCT reported a lower likelihood of menarche during the study period in the intervention group.

The two prospective athlete/nonathlete studies found differences in AAM that were of similar size to those athlete/nonathlete studies that recalled AAM, which reduces the likelihood of substantial recall bias. Therefore, the main limitation of these studies, as in all the other types of study considered, is the possibility of confounding due to other differences in behaviour or other attributes between athletes and nonathletes. Across all studies, only one (the PCS by Merzenich et al.) attempted to control for differences in diet, finding a significant positive association between PA duration and menarche 22. Even the RCT provides limited evidence for a direct effect of PA on menarche timing because it tested a multicomponent intervention that targeted diet as well as PA. *Post hoc* modelling by those study authors estimated that change in PA accounted for 1/3 of the intervention effect on menarche timing 18.

Other limitations of the included studies deserve mention and are informative for the design of future studies. Associations with energy expenditure are highly likely to be confounded by the established strong relationships between body weight and total energy expenditure (positive), and between body weight and AAM (negative). Therefore, in studies of energy expenditure, adjustment for body weight is essential. Cohort studies (one PCS and two HCS) that tested binary PA groupings failed to describe the criteria used to define these groupings 23, 27, 28. Finally, it is important to highlight the appreciable error in estimating PA. No study used an objective measure of PA, and the limitations of self‐reported PA, for example social desirability bias, are well‐recognised. Merzenich et al. 22 reported a correlation coefficient of only 0.3 between energy expenditure assessed by questionnaire versus by seven‐day diary. To improve reliability, they restricted their analysis to individuals who reported consistent PA durations between instruments, also their analysis controlled for the relevant confounders diet and per cent body fat, and they found a significant association between PA duration and timing of menarche 22. Moisan et al. 19 found no significant association in analyses adjusting only for age and mother's AAM. Both studies categorised PA duration into quartiles 19, 22, but these groups might not be directly comparable, and they were generated from different populations (Germany, Canada). Finally, there was considerable variation in exposure assessments, populations considered and statistical analyses. Furthermore, even within groups of relatively consistent study design and exposure, different analytical strategies (i.e. categorisation of exposure status, choice of statistical model and adjustment for confounders) precluded direct comparison of effect estimates. As a result, meta‐analysis was determined to be neither feasible nor appropriate.

It has been proposed that the effect of PA on puberty timing is mediated by body composition, specifically adipose tissue 30. The adipocyte‐secreted hormone, leptin, is known to stimulate the reproductive hormone axis and trigger reproductive maturation in women 25. Conversely, low leptin levels may mediate the effects of negative energy balance on delaying/disrupting menstruation via suppression of GnRH pulsatility 31. In this regard, nutritional restriction is a key potential confounder in the athlete/nonathlete studies, as thinness may confer advantages in several sports 32. Additionally, a selection effect, whereby individuals predisposed to thinness and later pubertal maturation are more likely to become and succeed as athletes, is also possible 32. However, the observation of significant differences in AAM in athletes engaged in sports not advantaged by thinness (e.g. swimming, basketball) indicates that nutritional deprivation is unlikely to explain the entirety of the observed associations 31, 33. Furthermore, some population‐based studies reported associations with PA that were independent of body weight. For example, Morris et al. 24 found that adjustment for childhood weight did not attenuate the association between PA and AAM. However, it is possible that changes in body composition (i.e. adiposity) occurred without impacting overall weight. Furthermore, in the identified RCT, change in PA appeared to mediate the intervention effect separately from changes in BMI or skinfold thickness 18. Additionally, the multivariate analysis reported by Merzenich et al. 22 revealed an independent effect of PA, when adjusted for percentage body fat. These results indicate that PA might exert some effects on AAM through pathways independent of adiposity.

In conclusion, this systematic review synthesises the published evidence for the association between PA and AAM. Our meta‐analysis indicates a significant delay in menarche, of roughly one year, in athletes compared to controls. Most studies were of elite athletes; however, studies of novice athletes also showed a significant delay of around half a year. Supportive evidence of an effect of PA on delaying menarche was identified from one RCT. However, lack of intervention specificity in the RCT, and confounding by diet and other factors, and potential large errors in PA measurement in the observational cohorts limit estimation of the true effect size. Overall, the strength of evidence based on reported general population samples is low. Future studies are needed to clarify the magnitude and nature of the effect of childhood PA on the timing of menarche in the general population.

## Funding

KKO and SB are supported by the Medical Research Council (Unit programmes: MC_UU_12015/2 and MC_UU_12015/3).

## Conflict of interest

No conflicts of interest.

## Supporting information


**Table S1** Population studies exclusion criteria on full text review.
**Table S2** Prospective cohort studies: summary of methods.
**Table S3** Historical cohort studies (cross sectional): summary of methods.
**Table S4** Prospective cohort population studies, quality assessment.
**Table S5** Cross‐sectional population studies, quality assessment.
**Table S6** Athlete/non‐athlete studies: summary of methods.
