# Supplementary material for: Systematic review and meta‐analysis of the association between childhood physical activity and age at menarche
Source: Acta Paediatr. 2019 Jan 15;108(6):1008–15. doi: 10.1111/apa.14711 (PMC6563453; doi:10.1111/apa.14711)
Supplement: Supplementary file 1 — Table S1 Population studies exclusion criteria on full text review. Table S2 Prospective cohort studies: summary of methods. Table S3 Historical cohort studies (cross sectional): summary of methods. Table S4 Prospective cohort population studies, quality assessment. Table S5 Cross‐sectional population studies, quality assessment. Table S6 Athlete/non‐athlete studies: summary of methods. [file APA-108-1008-s001.docx]

**Supporting Information**

Table S1.A. Population Studies Exclusion Criteria on Full Text Review

| **Exclusion Criteria** | **Number of Studies Excluded** |
| --- | --- |
| A. Does not report a measure of the association between physical activity and age at menarche | n=15 |
| B. Tests the association in the opposite direction (i.e. the impact of puberty timing on subsequent physical activity) | n=31 |
| C. Physical activity measure not specified as prior to puberty | n=14 |
| E. More appropriate for Athlete versus Non-athlete studies | n=1 |

Table S1.B. Athlete versus Non-athlete Studies Exclusion Criteria on Full Text Review

| **Exclusion Criteria** | **Number of Studies Excluded** |
| --- | --- |
| A. Did not report age at menarche | n=7 |
| B. Physical activity measure not specified as prior to menarche | n=3 |
| C. Review article without original data | n=4 |
| D. Duplicate publication | n=1 |
| E. No comparison to non-athlete | n=12 |
| F. Not accessible | n=6 |

Table S2. Prospective Cohort Studies: Summary of Methods

| **First author (citation)** | **Country,**  **Year Published** | **Sample Size** | **Sampling Method** | **PA Measure** | **PA categories** | **Follow-Up** |
| --- | --- | --- | --- | --- | --- | --- |
| 1) Tehrani et al. (23) | Iran, 2014 | n=402 | Random sample of urban population | Binary (active vs. passive), based on reported activities |  | 3 years |
| 2) Koo et al. (21) | Canada, 2002 | n=637 | Daughters of another study | Energy expenditure (kcal/week), from a habitual PA questionnaire | - Quartile 1: <1943.6 - Quartile 2: 1943.6- - 3724.9 - Quartile 3: 3724.9-6191.3 - Quartile 4: >.6191.3 | 3 years |
| 3) Merzenich et al. (22) | Germany, 1993 | n=167 | Random population-based sample | Energy expenditure (kj/kg), from a habitual PA questionnaire; also 7-day PA diary |  | 2 years |
| 4) Moisan et al. (19) | Canada, 1991 | n=2,487 | Convenience sample (122/178) of French-speaking public schools in Quebec City | Energy expenditure (not defined), from 7-day recall |  | 2 years |
| 5) Chie et al. (20) | Taiwan, 1997 | n=799 | Fourth grade girls in 8 elementary schools | Energy expenditure (kcal/week), from questionnaire | Ordered categories   - <2000 - 2,000-3,999 - >4,000 | 1 year |
| 6) Chavarro et al. (18)  -Randomised Control Trial | United States,  2005 | n=422 | 6th and 7th grade girls from 10 schools in 4 communities (4 pairs matched for town, 1 pair matched for size and race/ethnic composition) | MVPA duration (h/week), from 16-item validated questionnaire |  | 2 years |

Abbreviations: MET: metabolic equivalent of task, MVPA: moderate to vigorous physical activity

Table S3. Historical Cohort Studies (Cross Sectional): Summary of Methods

| **First author (citation)** | **Country,**  **Year Published** | **Sample Size** | **Sampling Method** | **PA Measure** |
| --- | --- | --- | --- | --- |
| Morris (24) | UK,  2010 | n=81,438 | Family-based breast cancer study | Recall of childhood PA level (questionnaire) |
| Papadimitriou (28) | Greece,  2008 | n=750 | High school girls from 6 Athens schools | Recall of premenarcheal participation in and duration of sports activities  Categorised as: “Active” or “Passive” (not defined) |
| Chavarro (25) | Colombia,  2004 | n=3,206 | Female undergraduate students at one university | Recall of premenarcheal average hours per day (questionnaire) |
| Vandeloo (27) | Belgium,  2007 | n=1,146 | First 115 children attending the second year of secondary school at 10 ‘well-distributed’ locations | Questionnaire recall of participation in sports.  Categorised as: “Some sport” or “No sport” (not defined) |
| Veronesi (26) | Italy,  1994 | n=2,930 | 3 nationally representative surveys of women and 1 survey of 5 secondary schools | Recall of premenarcheal participation in and duration of sports activities.  Categorized as: “Moderate” (2 hrs/week), “Regular” (5-6 hrs/week), or “Intense” (competitions or >6hrs/week) |

Table S4. Prospective Cohort Population Studies, Quality Assessment

| **Criteria** | **1) Tehrani** | **2) Koo** | **3) Merzenich** | **4) Moisan** | **4a) Moisan** | **5) Chie** |
| --- | --- | --- | --- | --- | --- | --- |
| 1. Was the research question or objective in this paper clearly stated? | Y | Y | Y | Y | Y | Y |
| 2. Was the study population clearly specified and defined? | Y | Y | Y | Y | Y | N |
| 3. Was the participation rate of eligible persons at least 50%? | Y | Y | Y | Y | NA | CD |
| 4. Were all the subjects selected or recruited from the same or similar populations (including the same time period)? Were inclusion and exclusion criteria for being in the study pre-specified and applied uniformly to all participants? | Y | Y | Y | Y | Y | Y |
| 5. Was a sample size justification, power description, or variance and effect estimates provided? | N | N | N | N | N | N |
| 6. For the analyses in this paper, were the exposure(s) of interest measured prior to the outcome(s) being measured? | Y | Y | Y | Y | Y | Y |
| 7. Was the timeframe sufficient so that one could reasonably expect to see an association between exposure and outcome if it existed? | Y | Y | Y | Y | NA | Y |
| 8. For exposures that can vary in amount or level, did the study examine different levels of the exposure as related to the outcome (e.g., categories of exposure, or exposure measured as continuous variable)? | N | Y | Y | Y | Y | Y |
| 9. Were the exposure measures (independent variables) clearly defined, valid, reliable, and implemented consistently across all study participants? | N | Y | N | Y | Y | CD |
| 10. Was the exposure(s) assessed more than once over time? | CD | N | Y | Y | N | N |
| 11. Were the outcome measures (dependent variables) clearly defined, valid, reliable, and implemented consistently across all study participants? | Y | Y | Y | Y | Y | Y |
| 12. Were the outcome assessors blinded to the exposure status of participants? | CD | NA | NA | NA | NA | NA |
| 13. Was loss to follow-up after baseline 20% or less? | CD | Y | CD | Y | NA | Y |
| 14. Were key potential confounding variables measured and adjusted statistically for their impact on the relationship between exposure(s) and outcome(s)? | Y | Y | Y | Y | N | N |
| Total | 8 | 11 | 10 | 12 | 7 | 7 |

Abbreviations: CD=cannot discern, NA=not applicable

Table S5. Cross-Sectional Population Studies, Quality Assessment

| **Criteria** | **1) Morris** | **2) Papadimitriou** | **3) Chavarro** | **4) Vandeloo** | **5) Veronesi** |
| --- | --- | --- | --- | --- | --- |
| 1. Was the research question or objective in this paper clearly stated? | Y | Y | Y | Y | Y |
| 2. Was the study population clearly specified and defined? | Y | Y | Y | Y | Y |
| 3. Was the participation rate of eligible persons at least 50%? | CD | Y | Y | Y | CD |
| 4. Were all the subjects selected or recruited from the same or similar populations (including the same time period)? Were inclusion and exclusion criteria for being in the study pre-specified and applied uniformly to all participants? | Y | Y | Y | CD | Y |
| 5. Was a sample size justification, power description, or variance and effect estimates provided? | N | Y | N | N | N |
| 6. For the analyses in this paper, were the exposure(s) of interest measured prior to the outcome(s) being measured? | N | N | N | N | N |
| 7. Was the timeframe sufficient so that one could reasonably expect to see an association between exposure and outcome if it existed? | NA | NA | NA | NA | NA |
| 8. For exposures that can vary in amount or level, did the study examine different levels of the exposure as related to the outcome (e.g., categories of exposure, or exposure measured as continuous variable)? | Y | N | Y | N | Y |
| 9. Were the exposure measures (independent variables) clearly defined, valid, reliable, and implemented consistently across all study participants? | N | N | N | CD | CD |
| 10. Was the exposure(s) assessed more than once over time? | N | N | N | N | N |
| 11. Were the outcome measures (dependent variables) clearly defined, valid, reliable, and implemented consistently across all study participants? | Y | Y | Y | Y | Y |
| 12. Were the outcome assessors blinded to the exposure status of participants? | NA | NA | NA | NA | NA |
| 13. Was loss to follow-up after baseline 20% or less? | NA | NA | NA | Y | NA |
| 14. Were key potential confounding variables measured and adjusted statistically for their impact on the relationship between exposure(s) and outcome(s)? | Y | N | Y | Y | N |
| Total | 6 | 6 | 7 | 6 | 5 |

Abbreviations: CD=cannot discern, NA=not applicable

Table S6. Athlete/non-athlete studies: Summary of Methods

| **Title (Author, Citation)** | **Country,**  **Year Published** | **Sample Size** | **Athletes Criteria** | **Athlete Status*** | **Controls** | **Study Design** |
| --- | --- | --- | --- | --- | --- | --- |
| Maimoun (34) | France  2011 | Athletes=23  Non-athletes=23 | **Gymnasts:**  Age: 10-17 yr  Training: 12-30 h/week  -starting age: 5.3 ± 1.3 yr | Elite | Age: matched  PA: <3 h/week | Cross-sectional |
| Donoso (35) | Spain  2010 | Athletes=22  Non-athletes=30 | **Ballet Dancers:**  Age: 11.3±0.8 yr (at baseline)  Training: >18  h/week | Elite | Age: matched  (matched: weight)  PA: <3 h/week | Prospective  (through puberty) |
| Schevchenko (29) | Ukraine  2008 | Athletes=130  Non-athletes=45 | **Gymnasts: (n=45)**  Training: 18-20 h/week  -starting age: 4.6 yr  **Badminton: (n=40)**  Training: 8-10 h/week  -starting age: 9.1 yr  **Trampoline: (n=45)**  Training: 10-12 h/week  -starting age: 6.2 yr  Age (all): 10-17 yr | Elite | Age: matched  PA: school physical education classes only | Cross-sectional |
| Matthews (36) | Australia  2006 | Athletes=82  Non-athletes=61 | **Dancers:**  Age: 8-11 yr (at baseline)  Training: 1-10 h/week  -starting age: 4.4 ± 1.5 yr | Novice | Age: matched  (matched: postcode)  PA: not stated | Prospective  (3 yr) |
| Torstveit (32) | Norway  2005 | Athletes=669  Non-athletes=607 | **National Athletes:**  Age: 13-39 yr  Training: 13.2 (SD=5.2) h/week  -66 different sports/events | Elite | Age: 13-19 yr  -random population sample  PA: 5.3 (SD=5.3) h/week | Cross-Sectional |
| Zanker (37) | UK  2004 | Athletes=18  Non-athletes=18 | **Gymnasts:**  Age: 20-32 yr (retired: 3-12 yrs.)  Training: 12-16 h/week  -starting age: 7±3 yrs. (>3 yr pre-menarche) | Elite | Age: matched  (matched: body mass, stature)  PA: school physical education classes only | Cross-Sectional, Retrospective |
| Muñoz (38) | Spain  2004 | Athletes=21  Non-athletes=14 | **Rhythmic Gymnasts: (n=9)**  Age: 14-18 yr  Training: >20 h/week  **Ballet Dancers: (n=12)**  Age:14-18 yr  Training: >20 h/week  -duration: >5 yr history of training | Elite | Age: matched  (matched: geographical area, social group)  PA: < 3h/week | Cross-Sectional |
| Klentrou (39) | Greece & Canada  2003 | Athletes=45  Non-athletes=78 | **Rhythmic Gymnasts:**  Age: 14-15 yrs.  Training: 5-36 h/ week  -duration: 3-9 consecutive years | Elite | Age: matched  PA: not part of a competitive sports team | Cross-Sectional |
| Lucas (40) | New Zealand  2003 | Athletes=15  Non-athletes=27 | **Runners:**  Age: 12-14 yrs.  Training: 1hr, 2-3 times/week  -duration: >2 yrs. | Novice | Age: 12-14 yrs.  PA: recreational sport, excluding running | Cross-Sectional |
| Dusek (33) | Croatia  2001 | Athletes=72  Non-athletes=96 | **Runners: (n=34)**  Training: 18±4 h/week  -starting age: 12.6±2.0 yr  **Volleyball: (n=10)**  Training: 19±3 h/week  -starting age: 10.7±1.1 yr  **Basketball: (n=18)**  Training: 18±0 h/week  -starting age: 12.6±1.3 yr  **Ballet: (n=10)**  Training: 18±4 h/week  -starting age: 6.0±0.5 yr  Age (all): 15-21 | Elite | Age: 15-21 yr  PA: school physical education classes only | Cross-Sectional,  Retrospective |
| Valentino (41) | Italy  2001 | Athletes=29  Non-athletes=30 | **Current Dancers: (n=20)**  Age: mean 21-22 yr  Training: >10 h/week  -duration: >10 yr  (started in preadolescence, at mean age =7.3 yr)  **Former Dancers: (n=9)**  Age: mean 21-22 yr  -duration: >10 yrs. (started in preadolescence, at mean age =7.3 yr) | Elite | Age: matched  PA: <2 h/week | Cross-Sectional,  Retrospective |
| Constantini (31) | Not stated  1995 | Athletes=69  Non-athletes=279 | **Swimmers:**  Age: 12-20 yrs.  Training: 3 sessions/week | Elite | Age: matched  PA: not stated | Cross-Sectional |
| Lindholm (42) | Sweden  1992 | Athletes=19  Non-athletes=21 | **Gymnasts:**  Age: 19-23 yrs.  Training: 10-20 h/week | Elite | Age: matched  PA: “only moderate PA in leisure time” | Cross-Sectional,  Retrospective |

*Elite was defined as meeting one of the following criteria: national level sports, special school for athletics, more than 10h/week, explicitly stated

Abbreviations: PA: Physical activity
